# Dietary Guanidinoacetic Acid Supplementation Improves Growth Performance of Plateau Yaks Through Plasma Metabolome Modulation

**DOI:** 10.3390/biology14111600

**Published:** 2025-11-15

**Authors:** Yinjie You, Li Zhang, Lin Fu, Xianwen Dong, Zhongli Peng, Yu Zeng, Gaofu Wang, Juncai Chen, Yanhua Gao, Jia Zhou

**Affiliations:** 1 Chongqing Academy of Animal Sciences, Chongqing 402460, China; 2 Bureau of Agricultural and Rural Affairs of Guangyuan City, Guangyuan 628017, China; 3 Key Laboratory of Animal Science of National Ethnic Affairs Commission of China, Ministry of Education Key Laboratory of Qinghai-Tibetan Plateau Animal Genetic Resources Reservation and Utilization, College of Animal and Veterinary Sciences, Southwest Minzu University, Chengdu 610041, China; 4 College of Animal Science and Technology, Southwest University, Chongqing 400715, China

**Keywords:** guanidinoacetic acid, yaks, growth performance, metabonomics

## Abstract

**Simple Summary:**

This study evaluated the effects of dietary guanidinoacetic acid (GAA) supplementation on growth and metabolism in yaks by randomly assigning twenty-four male animals to a control group (basal diet) and two treatment groups (0.055% or 0.11% GAA) for a 90-day feeding trial. The results showed a positive trend in growth performance, with the higher GAA supplementation level leading to a more noticeable improvement in average daily gain. Serum biochemical indices were generally unaffected, though a tendency of reduced glutathione peroxidase activity was observed. Non-targeted metabolomics in plasma identified multiple differentially abundant metabolites across groups. Enrichment analysis highlighted significant alterations in metabolic pathways related to tryptophan, glycerophospholipid, and arginine metabolism. Notably, metabolites such as N(omega)-Hydroxyarginine, 5-hydroxytryptophan, and serotonin were specifically affected. In conclusion, dietary GAA supplementation, particularly at 0.11%, has the potential to enhance growth in yaks, which may be attributed to its role in modulating amino acid metabolism, especially arginine and tryptophan pathways. These findings provide a metabolic perspective for the application of GAA as a feed additive in yak production.

**Abstract:**

This study aimed to investigate the effects of dietary guanidinoacetic acid (GAA) supplementation on yak physiology by evaluating growth performance, serum biochemical indices and plasm metabolomic profiles to elucidate the underlying regulatory mechanisms. Twenty-four male yaks (4–5 years; 249.38 ± 11.69 kg BW) were randomly allocated to three dietary treatments (*n* = 8): CON (basal diet), GAA1 (basal diet + 0.055% GAA), and GAA2 (basal diet + 0.11% GAA), with 55:45 of concentrate:roughage (DM basis). After a 10-day adaptation period, the feeding trial lasted 90 days. Body weights were measured on days 0 and 90 for growth performance evaluation, with blood samples collected on the final day for separation into serum and plasma to assess serum metabolic and antioxidant parameters and for plasma metabolomic profiling. The result showed that growth performance parameters displayed a positive trend, with average daily gain (ADG) showing marginal improvement (*p* = 0.072). Serum biochemical analysis revealed that dietary supplementation of GAA had no effect on serum biochemical parameters while tendency decreased GSH-Px activity (*p* = 0.087). Non-targeted metabolomics identified 39–121 differentially abundant metabolites in plasma across treatment groups. Kyoto encyclopedia of genes and genomes (KEGG) enrichment analysis of these metabolites revealed pathways such as tryptophan metabolism, glycerophospholipid metabolism, and arginine metabolism. Among the differentially abundant metabolites, N(omega)-Hydroxyarginine and tryptophan metabolites such as 5-hydroxytryptophan and serotonin were specifically highlighted. In conclusion, dietary supplementation of GAA in yaks has been confirmed to improve ADG, with a 0.11% supplementation level being more effective, and this may be associated with GAA enhancing amino acid metabolism, particularly arginine and tryptophan metabolism.

## 1. Introduction

Yaks (*Bos grunniens*), as the dominant livestock species on the Qinghai-Tibetan Plateau, provide multiple essential resources including meat, milk, and fur, serving as crucial livelihood and economic assets for Tibetan pastoral communities [1]. Natural pasture-fed yaks in the extreme plateau environment suffer from nutritional deficits due to extended cold periods (exceeding 180 days/year) and limited forage growth windows, resulting in insufficient biomass yield and inconsistent nutrient profiles that cannot satisfy yaks’ metabolic requirements through grazing alone [2]. Consequently, the distinctive seasonal body weight dynamics (warm-season accumulation/cold-season depletion) in pasture-fed yaks lead to growth retardation, extended fattening duration, deteriorated meat characteristics, and significant financial impacts on yak farming operations [3]. Furthermore, more than half of the grassland in Qinghai-Tibetan plateau being overgrazed for almost half a century had led to accelerated grassland degradation. Correspondingly, the feeding patterns of yaks had gradually transferred from extensive grazing to intensive house feeding. Concurrently with China’s growing beef consumption standards, there is an urgent need to refine dietary formulations and develop effective feed additives to enhance yak growth performance and meat quality.

Creatine is synthesized endogenously from arginine, glycine and methionine, serves as a critical energy buffer in animal musculature and neural tissues by facilitating rapid aenosine tiphosphate (ATP) regeneration [4]. However, endogenous creatine production satisfies only approximately 50% of physiological requirements, necessitating dietary supplementation [5]. Due to its relative instability and lack of regulatory approval as a feed additive [6], creatine has been largely replaced by its metabolic precursor guanidinoacetic acid (GAA), which is now widely adopted in livestock and poultry nutrition as a stable, cost-effective, and well-documented safe alternative for creatine supplementation [7,8,9]. Supplemental GAA has been shown to positively influence carcass composition and meat quality in growing-finishing pigs [10] and to enhance both growth metrics and meat attributes in poultry [11]. However, while these monogastric benefits are well-documented, research on GAA supplementation in ruminant systems remains preliminary, particularly concerning ideal inclusion rates, delivery methods, and underlying physiological mechanisms.

As a powerful means of uncovering how dietary nutrition influences animal metabolic pathways, blood metabolomics employs high-throughput technologies to measure changes in the blood metabolome. It also serves as a reliable tool for evaluating the alterations in yak body condition following concentrate supplementation in both cold and warm seasons [12,13]. We hypothesized that dietary supplementation with GAA would alter the blood metabolite profile of yaks on the plateau. Accordingly, we analyzed growth performance, serum biochemical and antioxidant parameters, and plasma metabolic profiles in yaks after 90 days of GAA supplementation. There, this study aims to elucidate the effects of dietary GAA supplementation on growth and blood parameters in yaks.

## 2. Materials and Methods

### 2.1. Animal Ethics Statement

All animal procedures were conducted in accordance with relevant ethical guidelines and were reviewed and approved by the Animal Welfare Committee of Southwest Minzu University (Approval No. P20220710-1).

### 2.2. Animals and Experimental Design

This experiment was conducted from July to November 2022 at the Tibetan Plateau Pastoral Co., Ltd. in Litang County (28.95° N, 99.32° E), Ganzi Tibetan Autonomous Prefecture, Sichuan Province. The study area, located at approximately 4000 m above sea level, features abundant sunshine, intense solar radiation and strong winds, with an annual average temperature of 5.9 °C and precipitation exceeding 700 mm. A total of 24 male yaks (4–5 year-old; initial BW 249.38 ± 11.69 kg) were assigned to three experimental groups (*n* = 8) based on the principle of similar BW randomization: CON receiving basal diet (55% concentrate and 45% roughage), GAA1 (basal diet + 0.055% GAA), and GAA2 (basal diet + 0.11% GAA), where GAA was precision-blended into the concentrate during feed manufacturing. GAA was purchased from Guangdong Xinnandu Feed Technology Co., Ltd. (Guangzhou, China) with a purity of ≥90%. The supplementation dosage was determined based on previous reports in beef cattle [14] and sheep [15]. Following a 10-day adaptation, yaks were fed experimental diets for 90 days in a 100-day trial. Yaks were individually housed and had ad libitum access to water. Feeding occurred twice daily at 8:00 and 17:00, with up to 10% residual feed. The complete nutritional composition is detailed in Table 1.

### 2.3. Growth Performance

On day 0 and day 90 of the trial, the body weight of each yak was measured and recorded before morning feeding using a ground scale (Shanghai Xiongheng Electronic Technology Co., Ltd., Shanghai, China). The average daily gain (ADG) was calculated based on the initial body weight (IBW) and final body weight (FBW). Feed provided to the yaks and the residual was recorded daily throughout the experiment and calculated the dry matter intake (DMI). The feed to gain ratio (F/G) was calculated based on the DMI and ADG.

### 2.4. Blood Sample Collection and Processing

On day 90 of the experiment, following an overnight fast, blood samples were collected from the jugular vein of five randomly selected yaks per group (with comparable BW) prior to morning feeding. For plasma preparation, 10 mL of blood was collected into anticoagulant vacuum blood collection tubes (Hebei Kang Weishi Medical Technology Co., Ltd., Shijiazhuang, China), gently inverted, and centrifuged at 1500× *g* for 15 min at 4 °C. Simultaneously, serum was prepared from blood collected in vacuum blood collection tubes (Kang Weishi) by allowing it to clot for 30 min prior to centrifugation. All resultant plasma and serum samples were immediately aliquoted, frozen on dry ice, and stored at −80 °C until analysis.

### 2.5. Serum Biochemical Parameters

Serum biochemical profiling was conducted using a Hitachi 3100 automated clinical chemistry analyzer (Hitachi Ltd., Tokyo, Japan) in accordance with manufacturer’s specifications. Following controlled thawing at 4 °C, the following parameters were quantitatively determined: alkaline phosphatase (ALP), triglycerides (TG), total cholesterol (TC), glucose (GLU), total protein (TP), blood urea nitrogen (BUN), and albumin (ALB). The globulin fraction (GLB) was subsequently calculated as the arithmetic difference between TP and ALB concentrations.

### 2.6. Serum Antioxidant Parameters

Serum antioxidant parameters, including total antioxidant capacity (T-AOC), glutathione peroxidase (GSH-Px) activity, total superoxide dismutase (T-SOD) activity, and malondialdehyde (MDA) content, were measured using commercial assay kits from Nanjing Jiancheng Bioengineering Institute (Nanjing, China), following the manufacturer’s instructions. Absorbance was determined with a microplate reader (Bio-Rad Laboratories, Inc., Hercules, CA, USA).

### 2.7. Metabolome Sequencing Data Processing and Analysis

100 μL of plasma was combined with 400 μL of ice-cold extraction solvent (acetonitrile: methanol = 1:1, *v*/*v*) containing 0.02 mg/mL L-2-chlorophenylalanine (internal standard) in a 1.5 mL microcentrifuge tube. The mixture was vigorously vortexed for 30 s followed by ultrasonic extraction at 5 °C and 40 kHz for 30 min to ensure complete metabolite solubilization. Then, samples were incubated at −20 °C for 30 min followed by high-speed centrifugation (13,000× *g*, 4 °C, 15 min) to pellet precipitated proteins. The supernatant was then concentrated via nitrogen evaporation and reconstituted in 100 μL of ice-cold acetonitrile/water (1:1, *v*/*v*) using ultrasonic-assisted dissolution (40 kHz, 5 °C) for 5 min. Finally, the samples were clarified by centrifugation (13,000× *g*, 4 °C) for 10 min before analytical processing. The supernatant was transferred to sample vials for Liquid chromatography-tandem mass spectrometry (LC-MS/MS) analysis. Pooled quality control (QC) samples were analyzed periodically to monitor system stability throughout the experiment. Chromatographic separation was achieved using a Thermo UHPLC-Q Exactive HF-X system (Thermo Scientific, Waltham, MA, USA) with an ACQUITY HSS T3 column (100 × 2.1 mm, 1.8 μm; Waters Corporation, Milford, MA, USA) maintained at 40 °C. The chromatographic separation was performed at 40 °C using a binary mobile phase system consisting of 0.1% formic acid in water-acetonitrile (95:5, *v*/*v*; solvent A) and 0.1% formic acid in acetonitrile-isopropanol-water (47.5:47.5:5, *v*/*v*; solvent B) delivered at a constant flow rate of 0.40 mL/min. Mass spectrometric detection utilized heated ESI in dual-polarity mode with the following parameters: ion source temperature 425 °C, sheath/auxiliary gas flows 50/13 arb, spray voltage ±3500 V, stepped collision energy (20–40–60 eV), mass resolutions of 60,000 (full scan) and 7500 (MS/MS).

The LC-MS raw data were processed using Progenesis QI (v2.3, Waters, Milford, MA, USA) to generate a three-dimensional data matrix, comprising sample information, metabolite identities, and MS response intensities. The data were then refined through removal of internal standards and analytical artifacts (including system noise, column bleed, and derivatization byproducts), followed by peak alignment and metabolite annotation against the Human Metabolome Database (HMDB, http://www.hmdb.ca/, accessed on 12 November 2025) and Metlin (https://metlin.scripps.edu/, accessed on 12 November 2025) reference databases. The data matrix was preprocessed by retaining metabolic features present in ≥80% of samples, imputing values below the detection limit with the minimum observed value and applying sum normalization to correct for technical variations. Quality control samples with relative standard deviation (RSD) >30% were excluded, followed by log10 transformation. Multivariate analysis was performed using the R package “ropls” (v1.6.2), including principal component analysis (PCA) and orthogonal least partial squares discriminant analysis (OPLS-DA). Metabolites with the Variable importance in the projection (VIP) > 1 and *p* < 0.05 (Student’s *t*-test) were considered significantly different. These differential metabolites were subsequently mapped to Kyoto Encyclopedia of Genes and Genomes (KEGG) pathways for functional classification and enrichment analysis using Python (version 3.12)’s “scipy.stats” package. The enrichment approach extended single-metabolite annotation to pathway-level analysis, identifying biological pathways most relevant to the experimental conditions through systematic evaluation of metabolite groups within functional nodes.

### 2.8. Data Analysis

The data were initially organized in Microsoft Excel (Version 2021; Microsoft Corporation, Redmond, WA, USA). Normality (Shapiro–Wilk test) and homogeneity of variances (Levene’s test) were then assessed. All datasets satisfied the assumptions of normal distribution and homoscedasticity. Consequently, the data are presented as mean ± standard error of the mean (SEM), and one-way analysis of variance (one-way ANOVA) was employed for intergroup comparisons. The model for the data was the following equation:

*Y_ij_* = *μ* + *T_i_* + *ε_ij_* (1)

where *Y_ij_* is the dependent variable, *μ* is the overall mean, *T_i_* is the fixed effect of treatment and *ε_ij_* is the random error.

Statistical analysis was performed using the yak per replicate as the experimental unit. Tukey’s multiple comparison test was conducted to identify statistical differences, with significance levels established at *p* < 0.05 and high significance at *p* < 0.01. When 0.05 ≤ *p* < 0.10, the results between the groups were considered to indicate a statistical trend. The graph was visualized using GraphPad Prism 9 (GraphPad Software, San Diego, CA, USA).

## 3. Results

### 3.1. Growth Performance

The effects of GAA supplementation on yak growth performance are presented in Table 2. Results indicated that ADG showed a tendency to increase (0.05 < *p* < 0.10) with higher levels of GAA supplementation. However, DMI, FBW, and F/G remained unaffected (*p* > 0.05) by the dietary treatments.

### 3.2. Serum Biochemical Parameters

No significant differences (*p* > 0.05) were detected in ALT, AST, TP, ALB, ALP, GLB, GLU, TC, TG, or BUN concentrations across groups (Table 3), suggesting that GAA supplementation did not influence these metabolic parameters.

### 3.3. Serum Antioxidant Parameters

As shown in Table 4, dietary GAA supplementation had no significant effects on serum antioxidant parameters in yaks, except for a decreasing trend (0.05 < *p* < 0.10) in GSH-Px activity.

### 3.4. Plasma Metabolic Profile

**Figure 1:**
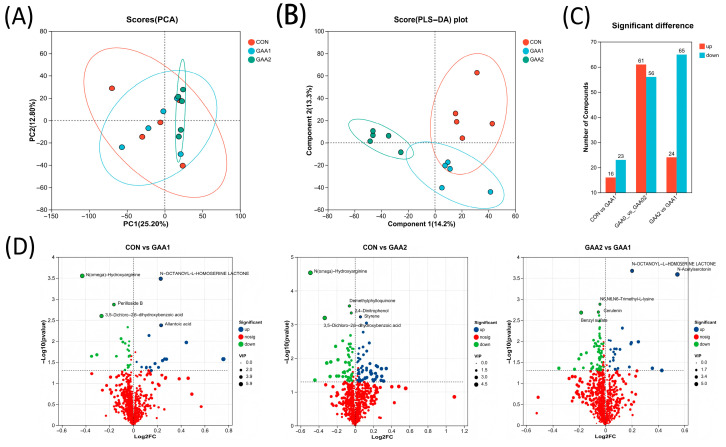
Multivariate analysis and differential metabolite analysis of the plasma metabolome. (**A**) Score plot from principal component analysis (PCA). (**B**) Score plot from orthogonal partial least squares-discriminant analysis (OPLS-DA). (**C**) Bar chart displaying the number of differential metabolites identified in each pairwise comparison. (**D**) Volcano plot illustrating the differential metabolites between groups. CON: basal diet; GAA1: basal diet supplemented with 0.055% guanidinoacetic acid; GAA2: basal diet supplemented with 0.11% guanidinoacetic acid.

As illustrated in Figure 1, distinct clustering patterns among the three treatment groups revealed significant alterations in yak plasma metabolic profiles induced by GAA supplementation. Untargeted LC-MS analysis with selection criteria of VIP ≥ 1 and *p* < 0.05 identified multiple differentially abundant metabolites. Compared to the CON group, the GAA1 group showed 16 downregulated and 23 upregulated metabolites, while the GAA2 group exhibited more substantial changes with 61 downregulated and 56 upregulated metabolites. Furthermore, comparative analysis between GAA2 and GAA1 groups demonstrated 65 downregulated and 24 upregulated metabolites, suggesting a dose-dependent metabolic response to GAA supplementation.

**Figure 2:**
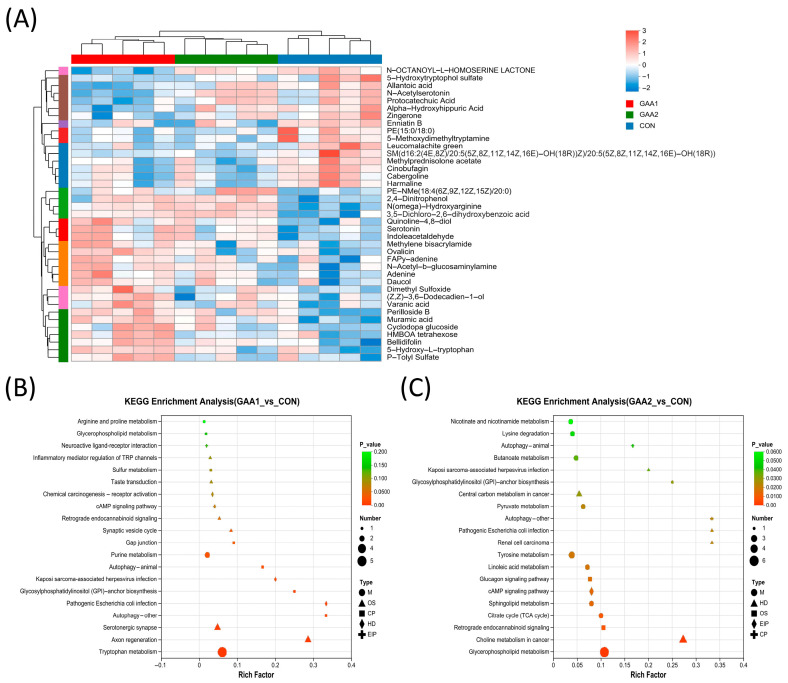
**Analysis of differential metabolites and functional enrichment.** (**A**) Heatmap displaying the top 50 differential metabolites identified in yak plasma among the three experimental groups. (**B**) KEGG pathway enrichment analysis of differential metabolites between the GAA1 and CON groups. (**C**) KEGG pathway enrichment analysis of differential metabolites between the GAA2 and CON groups. CON: basal diet; GAA1: basal diet supplemented with 0.055% guanidinoacetic acid; GAA2: basal diet supplemented with 0.11% guanidinoacetic acid.

Comparative metabolomic analysis revealed significant differences in plasma metabolite profiles between the GAA1 and CON groups and between the GAA2 and CON groups (Figure 2A). Compared to the CON group, the relative concentrations of N(omega)-Hydroxyarginine, 3,5-Dichloro-2,6-dihydroxybenzoic acid, Perilloside B, cyclodopa glucoside, Dimethyl sulfoxide, Serotonin, Bellidifolin, Indoleacetaldehyde in group GAA1 were upregulated, while the relative concentrations of N-OCTANOYL-L-HOMOSERINE LACONE, PE (15:0/18:0), SM (d16:2 (4E,8Z)/20:5(5Z,8Z,11Z,14Z,16E)-OH(18R)), Leucomalachite green, Protocatechuic acid, 5-Methoxydimethytryptamine, Zingerone, 5-hydroxytryptopgol sulfate, Enniatin B, Allantoic acid, N-Acetylserotonin, Alpha-Hydroxyhippuric acid in GAA1 were downregulated. Compared to CON group, the relative concentrations of dexpanthenol, ansamitocin P-3, Cer (d 18:2(4E,14Z)/TXB2), PE (16:1(9Z)/20:1(11Z)), Paxilline, SM (d16:2(4E,8Z)/20):5(5Z,8Z,11Z,14Z,16E)-OH(18R)), Enniatin B, Linalool (8-hydroxydihydro-), Cinncassiol D2 glucoside, 3-(Aminomethyl)-2,5,9-trimethyl-7H-furo(3,2-g)chromen-7-one, 5-Methoxydimethyltryptamine in group GAA2 were downregulated, and N(omega)-Hydroxyarginine, 3,5-Dichloro-2,6-dihydroxybenzoic acid, Tulathromycin A, PE (20:0/PGJ2), PC (14:0/20:5(5Z,8Z,11Z,14Z,17Z)), SM (D18:2(4E,14Z)/23:0), Lpetine I, PC (20:4(8Z,11Z,14Z,17Z)/18:0), PC (18:2(9Z,12Z)/18:1(11Z)) in group GAA2 were upregulated.

Comparative metabolic pathway analysis was performed to characterize the differential metabolic profiles in yak plasma among the CON, GAA1, and GAA2 groups. As shown in Figure 2B (GAA1 vs. CON) and Figure 2C (GAA2 vs. CON), KEGG enrichment analysis revealed distinct pathway alterations between groups. Specifically, the GAA1 group showed significant enrichment in tryptophan metabolism, axon regeneration, serotonergic synapse, pathogenic Escherichia coli infection, autophagy-related pathways, and glycosylphosphatidylinositol (GPI)-anchor biosynthesis compared to controls. In contrast, the GAA2 group exhibited predominant alterations in glycerophospholipid metabolism, choline metabolism in cancer, citrate cycle (TCA cycle), sphingolipid metabolism, cAMP signaling pathway, glucagon signaling pathway, and tyrosine metabolism.

### 3.5. Critical Plasma Metabolites

**Figure 3:**
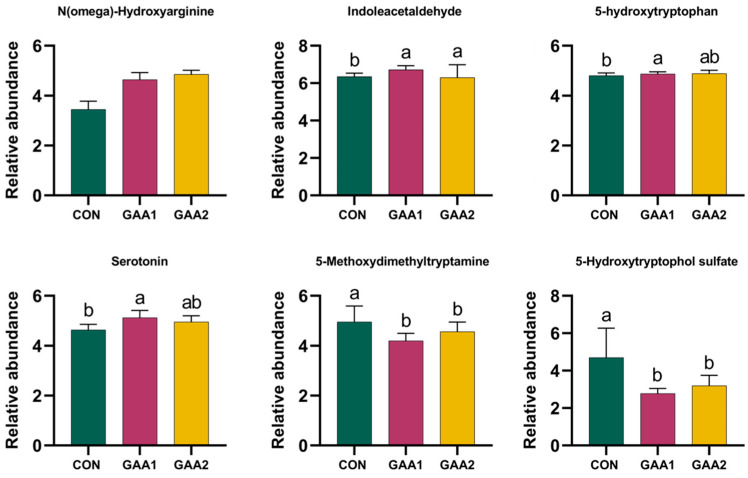
Effect of dietary guanidinoacetic acid supplementation on relative abundance of key metabolites in yak plasma. Data are presented as mean ± SD. Bars with different lowercase letters indicate significant differences (*p* < 0.05).

As shown in Figure 3, compared with the control (CON) group, dietary GAA supplementation significantly increased (*p* < 0.05) the relative abundances of N (omega)-Hydroxyarginine and indole acetaldehyde in yak plasma, while it decreased (*p* < 0.05) the levels of 5-hydroxytryptophol sulfate and 5-methoxydimethyltryptamine. Additionally, the relative abundances of 5-hydroxytryptophan and serotonin were significantly higher (*p* < 0.05) in the GAA1 group than in the CON group.

## 4. Discussion

As an endogenous amino acid derivative, GAA serves as an optimal creatine alternative owing to its favorable safety profile, controllable metabolism, facile synthesis, stable storage characteristics, and cost-effectiveness [16]. GAA acts as an arginine-sparing agent, which increases the supply of arginine available for critical physiological functions such as protein synthesis, nitric oxide production, and growth hormone secretion [6]. Furthermore, upon conversion to creatine, it enhances cellular energy metabolism by improving energy homeostasis, stimulating protein anabolism, and bolstering antioxidant defenses. Collectively, these actions ultimately lead to enhanced growth performance and productivity in animals [17]. In the present study, the ADG of yaks tended to increase with increasing GAA supplementation, which is consistent with previous studies in broilers [18], finishing pigs [10] and bulls [19]. Thus, dietary GAA showed a positive trend for improving growth performance in plateau yaks maintained in a housing system.

Serum biochemical indicators bridge dietary nutrient intake and physiological responses, offering a crucial window for assessing an animal’s overall health and metabolic status [20]. To evaluate the effects of dietary GAA on the nutritional metabolism of plateau yaks, this study quantified key serum parameters associated with nitrogen (TP, ALB and BUN), lipid (TG and TC), and carbohydrate (GLU) metabolism. In agreement with prior findings [5,21], dietary supplementation of GAA did not significantly alter the serum biochemical profile of yak bulls in the present study. The cooperative regulation of key physiological and antioxidant defense pathways by serum reactive oxygen species (ROS) and the antioxidant system hinges on a robust mechanism comprising enzymatic and non-enzymatic pathways that maintain a dynamic balance between ROS generation and clearance [22]. Contrary to reports in Hu sheep where GAA improved antioxidant status [23], dietary GAA supplementation in this study showed no significant effects on overall serum antioxidant parameters in yaks, except for a decreasing trend in GSH-Px activity. The lower supplemental dosage used herein likely accounts for this difference. These findings demonstrate that dietary GAA supplementation did not disrupt the overall metabolic and oxidative homeostasis in yaks.

The untargeted metabolomics results revealed that the differential metabolites identified in the plasma among the three groups were mainly enriched in amino acid and phospholipid metabolism pathways, reflecting corresponding shifts in the body’s overall metabolic status. Lipid metabolism is a complex biochemical reaction process that involves the synthesis, breakdown, and transport of substances such as fats, phospholipids, and cholesterol to maintain overall energy homeostasis in yaks [24]. Dietary supplementation with GAA can enhance energy metabolism by increasing phosphocreatine levels, while also stimulating fat utilization for energy production [25]. Factors that affect creatine supply or synthesis may deplete amino acid precursors such as arginine and glycine, thereby reducing the availability of free amino acids for protein synthesis and muscle growth [6]. In contrast, supplementing with GAA as a creatine precursor can alleviate the burden on amino acid metabolism imposed by endogenous creatine synthesis, allowing more arginine and glycine to be prioritized for growth and other physiological functions [26]. In this study, besides changes in BW, GAA supplementation also increased the plasma level of N(omega)-Hydroxyarginine. N(omega)-Hydroxyarginine is the optimal substrate for nitric oxide synthases [27], and exogenous supplementation of GAA can also influence the growth-promoting physiological functions mediated by nitric oxide [28]. In addition to arginine, alterations were detected in certain metabolites of the tryptophan metabolic pathway in this study. In mammals, the essential amino acid tryptophan is converted to 5-hydroxytryptophan (5-HTP) and then decarboxylated into 5-hydroxytryptamine (commonly known as serotonin), which subsequently executes important physiological functions [29]. In this process, indoleacetaldehyde and 5-hydroxytryptophol sulfate are metabolites of tryptophan and serotonin, respectively [30,31]. In this study, both 5-HTP and serotonin levels were higher in the GAA-supplemented group than in the control group. Meanwhile, indoleacetaldehyde was significantly upregulated while 5-hydroxytryptophol sulfate was significantly downregulated. These findings indicate that dietary GAA supplementation promotes the conversion of tryptophan to serotonin. Furthermore, dietary 5-HTP supplementation increased plasma serotonin level in Holstein steers and restored the reduced feed intake induced by ergot alkaloid poisoning [32]. Therefore, the results of the non-targeted metabolomics indicate that dietary GAA supplementation is beneficial for promoting amino acid metabolism in yaks, particularly the metabolism of arginine and tryptophan. However, as this interpretation relies on inferred rather than targeted metabolic evidence, it remains speculative and warrants further validation through dedicated analysis of key intermediates and related enzymes.

## 5. Conclusions

In conclusion, dietary GAA supplementation effectively enhanced the growth performance of yaks, with the effects showing a dose-dependent improvement. Plasma non-targeted metabolomics revealed that this beneficial effect may be associated with the promotion of amino acid metabolism. Despite the observed trend of improved ADG at the 0.11% level, further study is necessary to delineate the complete optimal dosage range and the effects of even higher supplementation levels. Nevertheless, these findings provide a scientific basis for the targeted application of GAA in nutritional strategies for high-altitude ruminants.

## Figures and Tables

**Table 1:** Ingredients and nutrient components of the basal diet (%, DM basis).

Diet Ingredients	Contents, %
Corn	40.15
Wheat bran	2.75
Soybean	8.80
Urea	0.22
NaCl	0.44
Premix ^1^	2.64
Whole maize silage	45.00
Total	100.00
Nutrient component ^2^	
NEmf, MJ/kg	6.35
Crude protein, %	12.88
Neutral detergent fiber, %	24.98
Acid detergent fiber, %	8.61
Crude ash, %	5.31
Total calcium, %	0.46
Phosphorus, %	0.40

^1^ The premix provided per kg of diet: 5000 IU of vitamin A, 550 IU of vitamin D, and 760 IU of vitamin E; along with the following trace minerals: 10 mg Cu, 50 mg Fe, 40 mg Mn, 40 mg Zn, 0.5 mg I, 0.2 mg Se, and 0.2 mg Co. ^2^ The NEmf was based on the Chinese Beef Cattle Feeding Standard (NY/T 815-2004), while all other values were measured.

**Table 2:** Effects of GAA supplementation on growth performance of yaks.

Item ^2^	Treatments ^1^			SEM	*p*-Value
	CON	GAA1	GAA2		
IBW (kg)	250.44	248.13	249.56	2.28	0.924
FBW (kg)	310.31	314.63	324.13	2.93	0.144
ADG (kg/d)	0.67	0.74	0.83	0.03	0.072
F/G	10.10	8.42	7.41	0.35	0.235
DMI (kg/d)	6.29	6.69	6.84	0.17	0.179

^1^ CON: basal diet; GAA1: basal diet supplemented with 0.055% guanidinoacetic acid; GAA2: basal diet supplemented with 0.11% guanidinoacetic acid. ^2^ IBW: initial body weight; FBW: final body weight; ADG: average daily gain; F/G: feed to gain ratio; DMI: dry matter intake.

**Table 3:** Effects of GAA supplementation on serum biochemical indexes.

Item ^2^	Treatments ^1^			SEM	*p*-Value
	CON	GAA1	GAA2		
TP (g/L)	88.80	92.46	90.26	1.32	0.557
ALB (g/L)	39.63	41.24	40.76	0.91	0.787
GLB (g/L)	49.16	51.22	49.50	1.07	0.734
ALP (U/L)	239.20	203.60	263.60	15.25	0.290
GLU (mmol/L)	5.94	5.10	4.93	0.30	0.364
TC (mmol/L)	1.48	2.25	2.22	0.21	0.246
TG (mmol/L)	0.35	0.38	0.32	0.03	0.778
BUN (mmol/L)	3.23	4.03	3.95	0.21	0.234

^1^ CON: basal diet; GAA1: basal diet supplemented with 0.055% guanidinoacetic acid; GAA2: basal diet supplemented with 0.11% guanidinoacetic acid. ^2^ TP, Total protein; ALB, Albumin; GLB, Globulin; ALP, Alkaline phosphatase; GLU, Glucose; TC, Total cholesterol; TG, Triglyceride; BUN, Blood urea nitrogen.

**Table 4:** Effects of GAA supplementation on serum antioxidant parameters.

Item ^2^	Treatments ^1^			SEM	*p*-Value
	CON	GAA1	GAA2		
T-AOC (mM)	0.21	0.18	0.20	0.02	0.765
GSH-Px (μmol/L)	211.83	124.70	126.26	41.25	0.087
T-SOD (U/mL)	239.10	253.57	238.47	14.96	0.562
MDA (nmol/mL)	3.07	3.17	3.20	0.37	0.314

^1^ CON: basal diet; GAA1: basal diet supplemented with 0.055% guanidinoacetic acid; GAA2: basal diet supplemented with 0.11% guanidinoacetic acid. ^2^ T-AOC, total antioxidant capacity; GSH-Px, glutathione peroxidase; T-SOD, total superoxide dismutase; MDA, malondialdehyde.

## Data Availability

All data generated or analyzed used in this study are available from the corresponding author upon request.
